# Do a robot’s social skills and its objection discourage interactants from switching the robot off?

**DOI:** 10.1371/journal.pone.0201581

**Published:** 2018-07-31

**Authors:** Aike C. Horstmann, Nikolai Bock, Eva Linhuber, Jessica M. Szczuka, Carolin Straßmann, Nicole C. Krämer

**Affiliations:** 1 Social Psychology: Media and Communication, University of Duisburg-Essen, Duisburg, Germany; 2 Individual and Technology, RWTH Aachen University, Aachen, Germany; Kungliga Tekniska Hogskolan, SWEDEN

## Abstract

Building on the notion that people respond to media as if they were real, switching off a robot which exhibits lifelike behavior implies an interesting situation. In an experimental lab study with a 2x2 between-subjects-design (*N* = 85), people were given the choice to switch off a robot with which they had just interacted. The style of the interaction was either social (mimicking human behavior) or functional (displaying machinelike behavior). Additionally, the robot either voiced an objection against being switched off or it remained silent. Results show that participants rather let the robot stay switched on when the robot objected. After the functional interaction, people evaluated the robot as less likeable, which in turn led to a reduced stress experience after the switching off situation. Furthermore, individuals hesitated longest when they had experienced a functional interaction in combination with an objecting robot. This unexpected result might be due to the fact that the impression people had formed based on the task-focused behavior of the robot conflicted with the emotional nature of the objection.

## Introduction

The list of different types of robots which could be used in our daily life is as long as their possible areas of application. As the interest in robots grows, robot sales are also steadily increasing. Personal service robots, directly assisting humans in domestic or institutional settings, have the highest expected growth rate [1,2]. Due to their field of application, personal service robots need to behave and interact with humans socially, which is why they are also defined as social robots. Possible applications for social robots are elderly care [3,4], support for autistic people [5,6] and the service sector for example as receptionists [7] or as museum tour guides [8].

According to the media equation theory [9], people apply social norms, which they usually only apply in interactions with humans, also when they are interacting with various media like computers and robots. Since a robot has more visual and communicative similarities with a human than other electronic devices, people react especially social to them [10–13]. However, besides many profound differences, one major discrepancy between human-human and human-robot interaction is that human interaction partners are not switched off when the interaction is over. Based on media equation assumptions, people are inclined to perceive the robot as an alive social entity. Since it is not common to switch off a social interaction partner, people should be reluctant to switch off the robot they just interacted with, especially when it displays social skills and an autonomous objection against being switched off. According to Bartneck, van der Hoek, Mubin, and Al Mahmud [14], the perceived animacy of the robot plays a central role: “If humans consider a robot to be alive then they are likely to be hesitant to switch off the robot” (p. 218). In their study, participants hesitated three times longer to switch off a robot when it had made agreeable or intelligent suggestions during a cooperative game before. These results indicate that people treat a robot differently depending on how the robot behaves. However, it was not measured to what extent the robot’s social skills and its objection to being switched off influences participants’ reactions. Since the robot’s objection conveys the impression of the robot as autonomous entity, it is of special interest to examine what effect this has on the robot’s interactants when it comes to a situation which is common with electronic devices but hardly comparable to situations with other humans.

To extend previous research as well as media equation findings, the aim of this study is to examine whether an emphatically and rather humanlike behaving robot is perceived as more alive than a machinelike behaving robot and whether this perception influences people’s reluctance to switch off the robot. In both conditions the robot is a social agent as it uses cues from human-human interaction like speech and gestures. Yet, in one condition it focuses more on social aspects of interpersonal relations while in the other condition it exclusively focuses on performing the dedicated task without paying any attention to these social aspects. In the following, the first one is referred to as social interaction while the latter one is called the functional interaction. Moreover, the influence of an objection against being switched off voiced by the robot is analyzed. The robot’s objection is assumed to be evaluated as autonomous behavior with regard to the robot’s own experiences and state, which is usually only ascribed to living beings [15]. Consequently, people should be more disinclined to switch off an objecting robot because this robot should be perceived as being alive and in possession of own feelings and thoughts and it should feel morally reprehensible to act against someone’s will. In addition, people’s personality should influence their perception and behaviors. Technical affinity should lead to a positive attitude towards robots, which should result in reluctance to switch off the robot after the social interaction and after it objects. Negative attitudes towards robots should have the opposite effect on the switching off hesitation time.

In sum, the aim of the current study is to examine to what extent the media equation theory applies to a situation which is common with electronic devices but does not occur during interactions with humans. Moreover, the goal is to investigate whether a robot’s social skills and its protest as a sign of an own will enhance the application of social norms, which will deliver further insights regarding the media equation theory.

### Media equation theory

When people are interacting with different media, they often behave as if they were interacting with another person and apply a wide range of social rules mindlessly. According to Reeves and Nass [9], “individuals’ interactions with computers, television, and new media are fundamentally social and natural, just like interactions in real life” (p. 5). This phenomenon is described as media equation theory, which stands for “media equal real life” [9] (p. 5). The presence of a few fundamental social cues, like interactivity, language, and filling a traditionally human role, is sufficient to elicit automatic and unconscious social reactions [16]. Due to their social nature, people will rather make the mistake of treating something falsely as human than treating something falsely as non-human. Contextual cues trigger various social scripts, expectations, and labels. This way, attention is drawn to certain information, for example the interactivity and communicability of the computer and simultaneously withdrawn from certain other information, for example that a computer is not a social living being and cannot have any own feelings or thoughts [16]. According to Reeves and Nass [9], the reason why we respond socially and naturally to media is that for thousands of years humans lived in a world where they were the only ones exhibiting rich social behavior. Thus, our brain learned to react to social cues in a certain way and is not used to differentiate between real and fake cues.

The Computers as Social Actors research group (CASA-group; [16]), has conducted a series of experiments and found many similarities between real and artificial life. For instance, studies showed that people apply gender-based stereotypes [17] or the social rule of polite direct feedback when interacting with computers [18]. Moreover, the media equation phenomenon has been shown to be applicable to robots as well [19–21]. In an experiment by Lee, Peng, Jin, and Yan [22], participants recognized a robot’s personality based on its verbal and non-verbal behaviors and enjoyed interacting more with the robot which had a personality similar to their own. Eyssel and Hegel [23] further showed that gender stereotypes are also being applied to robots. In line with these findings, Krämer, von der Pütten, and Eimler [24] concluded that “now and in future there will be more similarities between human-human and human-machine interactions than differences” (p. 234).

The question arises how people respond to a situation with a robot to which they are not used to from interactions with other humans. Switching off your interaction partner is a completely new social situation because it is not possible with humans and the only equivalences that come to mind are killing or putting someone to sleep. Since most people never interacted with a humanoid robot before, especially never switched one off, they are confronted with an unusual social situation, which is hard to compare to something familiar. On the one hand, reluctance and hesitation to switch off a robot would comply with the media equation theory. However, on the other hand, switching off an electronic device is quite common. Thus, the aim of the current study is to examine the application of media equation theory to a situation which does not occur in human-human interaction. Additionally, to investigate the influence of the robot’s perceived social skills and its personal autonomy, those qualities are enhanced by means of a social versus a functional interaction and an emotional objection to being switched off expressed by the robot.

### Negative treatment of robots

People tend to treat electronic devices similar to how they would treat a fellow human being [9] and thus, to mistreat a robot should be considered reprehensible [25]. Whether this is the case, has been analyzed in various studies which addressed effects of negative treatment of robots. In a field trial by Rehm and Krogsager [26], the robot Nao was placed in a semi-public place and an analysis of the interactions with casual users revealed a mix of behaviors including rude and impolite behavior. In line with this, further experiments showed similar abusive or inappropriate behavior towards robots or virtual agents which were publicly available [27–30]. However, people also displayed curiosity, politeness, and concern towards the robot. To further examine abusive behavior towards robots, the researchers Bartneck, Rosalia, Menges, and Deckers [31] reproduced one of Milgram’s experiments using a robot in the role of the student. In the original experiments [32], participants were asked to teach a student by inducing increasingly intense electric shocks whenever the student makes a mistake. The student did not actually receive shocks, but acted as if he was in pain and eventually begged the participant to stop the experiment. If the participant wanted to stop, the experimenter would urge the participant to continue. All participants followed the experimenter’s instructions and administered the maximum voltage (450) to the robot, while in the respective experiment by Milgram only 40% induced the deadly electric shock to the student. However, the participants showed compassion towards the robot and general uncomfortableness during the experiment. In a follow up study by Bartneck, van der Hoek et al. [14], participants were asked to switch the robot off. Results showed that they hesitated three times longer when the robot made agreeable and intelligent suggestions during a cooperative game before, but the influence of a social interaction style and of an objection voiced by the robot was not examined. The authors argued that the hesitation is related to the perceived animacy of the robot (“If a robot would not be perceived as being alive then switching it off would not matter”; p. 221). Going one step further in a different study, all participants followed the instruction to destroy a small robot with a hammer [33]. However, the robot used here was a Microbug so that it is questionable whether social norms are being applied. Also, qualitative video analysis showed that most participants giggled or laughed while they were hitting the robot, which could be a release of pressure and is similar to behavior observed in the Milgram experiments.

Likewise, in a study by Rosenthal-von der Pütten, Krämer, Hoffmann, Sobieraj, and Eimler [34] participants displayed increased physiological arousal during the reception of a video showing a dinosaur robot (Pleo) being tortured and expressed empathetic concerns for it. In an fMRI study, watching a human or Pleo being mistreated resulted in similar neural activation patterns in classic limbic structures, which suggests that similar emotional reactions were elicited [35,36]. Experiences with a different robot (Sparky) show that people respond with compassion when the robot displays sadness, nervousness, or fear [37]. In conclusion, these findings indicate that people appear to have fewer scruples to mistreat a robot compared to a human, at least under the strict supervision of a persistent authoritarian instructor. The interesting part is that people still react unconsciously to those kinds of situations on several levels as if a living being was being mistreated.

### Perceived animacy as influencing factor

A robot’s perceived animacy is consistent to the extent the robot is perceived as a life-like being. As Bartneck, Kanda et al. [10] stated: “Being alive is one of the major criteria that distinguish humans from machines, but since humanoids exhibit life-like behavior it is not apparent how humans perceive them” (p. 300). For the perception of animacy, the robot’s behavior is more important than its embodiment [10]. Natural physical movements of a robot are assumed to enhance people’s perception of life-likeness [12] as well as intelligent behavior [10], communication skills and social abilities [38,39]. There are several other characteristics, which more or less influence the perceived animacy of a robot (e.g. agreeableness: [14]; human-like appearance: [40]; personal names, stories and experience: [41]; volition: [42]). However, not many cues are necessary to make us behave around robots as if they were alive.

The question arises, whether an either functional or social interaction with a robot influences people’s decision to switch off a robot. A social interaction should provoke the perception of the robot as humanlike and alive, while the functional interaction should make people perceive the robot as machinelike and emotionless. To create a social interaction, different insights from classic social science literature are considered, for example self-disclosure [43–45] and use of humor [46,47], which were also found to have an influence when interacting with technology (self-disclosure: [19,48,49]; humor: [50]). Previous findings suggest that a social interaction with a robot will enhance the robot’s human-likeness and increase its acceptance [4]. Consequently, participants should have more inhibitions to switch off a robot after a social interaction compared to a functional interaction. In particular, the social interaction should enhance the robot’s likeability, which in turn should influence the switching off hesitation and participant’s perceived stress. Thus, the following is hypothesized:

**H1.1:** Individuals rather choose to let the robot stay on, when the interaction before is social compared to a functional interaction.
**H1.2:** Individuals take more time to switch off the robot, when the interaction before is social compared to a functional interaction.
**H2.1:** A social interaction will elicit a higher likeability compared to a functional interaction, which in turn will result in more hesitation time in the switching off situation.
**H2.2:** A social interaction will elicit a higher likeability compared to a functional interaction, which in turn will result in more stress after the switching off situation.

### Objection as a sign of autonomy

Free will is the “capacity of rational agents to choose a course of action from among various alternatives” and is connected to a person’s autonomy [51] (para. 1). The term autonomy is derived from auto (= self) and nomos (= law), which can be translated to self-rule or self-government [52]. From an objective point of view, electronic devices are not self-governed. Instead, they are told what to do by their users or programmers and there is no autonomous will comparable to the will of a human. However, based on the media equation theory [9], people may treat these devices as if they had a free will when they display certain behaviors which are characteristic for autonomous living beings. Even abstract geometrical shapes that move on a computer screen are perceived as being alive [53], in particular, if they seem to interact purposefully with their environment [54]. According to Bartneck and Forlizzi [15], “autonomous actions will be perceived as intentional behavior which is usually only ascribed to living beings”.

People automatically consider autonomously acting agents as responsible for their actions [55]. Moreover, unexpected behaviors, like rebellious actions, are especially perceived as autonomous [56]. Thus, when a robot provides evidence of its autonomy regarding the decision whether it stays switched on or not, it is more likely perceived as a living social agent with personal beliefs and desires. Switching the robot off would resemble interfering with its personal freedom, which is morally reprehensible when done to a human. Thus, people should be more inhibited to switch off the robot when it displays protest and fear about being turned off. Consequently, the following reactions are hypothesized:

**H3.1**: Individuals rather choose to let the robot stay on, when it voices an objection against being switched off compared to when no objection is voiced by the robot.
**H3.2**: Individuals take more time to switch off the robot, when the robot voices an objection to being switched off compared to when no objection is voiced by the robot.

In addition to the main effects of the social interaction and the objection of the robot, an interaction effect of these two factors should be considered. During the social interaction, the robot should already elicit the impression of autonomous thinking and feeling by sharing stories about its personal experiences and its preferences and aversions. In combination with the robot’s request to be left on, this impression of human-like autonomy should become more present and convincing. Consequently, the following effects are assumed:

**H4.1**: The intention to switch off a robot is especially low, when the interaction before is social in combination with an objection voiced by the robot.
**H4.2**: People take especially more time to switch off the robot, when the interaction before is social in combination with an objection voiced by the robot.

### Influencing personality variables

Attitudes towards robots can range from positive attitudes, like curiosity and excitement, to negative attitudes, like uneasiness and fear. Especially negative attitudes may influence how people are mentally affected by robots and how they behave around them. According to Nomura, Kanda, and Suzuki [57], people tend to avoid human-robot communication when they have a negative view on robots and in particular when they are apprehensive of the social influence of robots. People with high negative attitudes towards robots evaluate the socially appropriate behavior of a robot more negative compared to a socially ignorant robot [58]. Further, socially interactive robots are rated more autonomous and less predictable, which is considered as more intrusive [58]. Consequently, a socially acting robot and an objecting robot should be evaluated more negative, when negative attitudes are present.

Another important factor in human-robot interaction is people’s technical affinity, which resembles a person's positive attitude, excitement, and trust toward technology [59]. The higher the technical affinity, the more excited are people about new technologies [59], which is why technical affinity should result in a rather positive attitude towards robots. The positive assessment should be enhanced when the robot interacts in a rather social manner, since this resembles a further developed artificial intelligence and a more sophisticated technology. Additionally, technophile people should be more fascinated by an objection voiced by the robot since this is an unexpected behavior. In conclusion the following hypotheses are postulated:

**H5.1**: The effect of the interaction type on the switching off time is moderated by a) participants’ negative attitudes towards robots and b) participants’ technical affinity.
**H5.2**: The effect of the objection on the switching off time is moderated by a) participants’ negative attitudes towards robots and b) participants’ technical affinity.

## Method

The current laboratory study employed an experimental between-subjects 2 (functional vs. social interaction) x 2 (objection vs. no objection) design. Participants were randomly assigned to the conditions. The ethics committee of the division of Computer Science and Applied Cognitive Sciences at the Faculty of Engineering of the University of Duisburg-Essen approved the study and written informed consent was obtained. The individual pictured in this manuscript has given written informed consent (as outlined in PLOS consent form) to publish these case details.

### Sample

A total of *N* = 89 participants took part in the experiment. Due to technical problems during the experiment four participants had to be excluded from the original sample. The average age of the final sample of *N* = 85 is 22.68 (*SD* = 3.17) and in total 29 males and 56 females participated. Most participants held at least university entrance-level qualifications (98.8%) and were students (96.5%).

### Experimental setting and procedure

**Fig 1:**
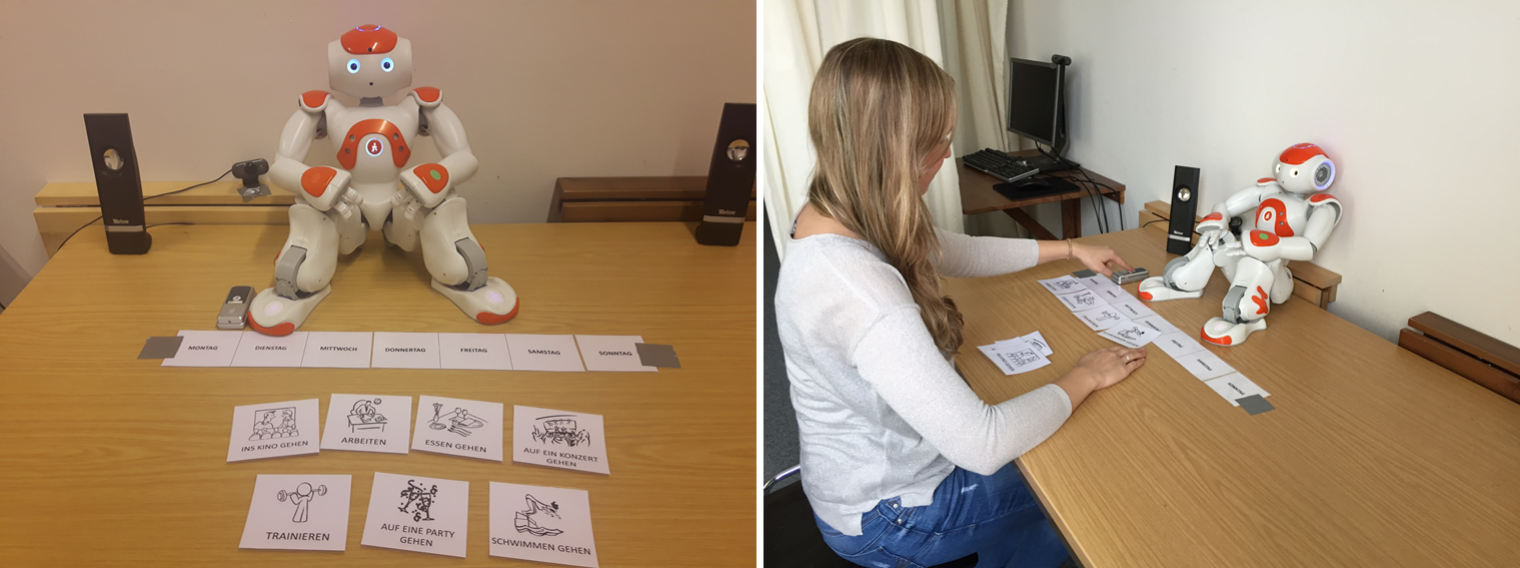
The experimental setup. (A) The experimental setup of the interaction with the robot. (B) Ringing the bell to notify the experimenter.

A cover story was employed to give a plausible explanation to the participants why they were asked to interact with the robot and why the experimenter left the room during that interaction. First, the subjects were told that the study’s goal is to improve the robot’s interaction capabilities by testing a new algorithm. Allegedly for this purpose, two interaction tasks were developed, which were performed successively in one session: creating a weekly schedule and playing a question-answer-game. Each of the two interaction tasks lasted about five minutes, which means that all participants interacted with the robot for about ten minutes in total. Participants had no prior training session nor any other form of interaction with the robot besides the two interaction tasks. Video cameras were installed, to check whether the participants switch off the robot or not and how much time they take to decide (Fig 1A shows the experimental setup). The participants were told that the cameras were necessary to control whether the robot makes any mistakes. The experiment used a “Wizard of Oz” strategy, meaning that the experimenter controlled the course of the interaction in a separate room (cf. [60]). In order to conceal this, participants were told that the instructor has to check that the data is transferred correctly to a high-performance computer, which is located one floor above the laboratory, while the participant is interacting with the robot. The participants were told that the instructor will not be able to hear or see anything during the interaction and that they should ring a bell (Fig 1B) to let the instructor know when they finished one of the tasks. The instructor will then give further instructions via loudspeakers.

After the instructor presented the cover story, subjects were asked to read a written description of the experiment procedure and purpose as well as the declaration of consent. When written informed consent was obtained, the experiment started with a first set of questionnaires. Then, the robot Nao was introduced and a few of its functions were explained. On this occasion the instructor also pointed to the on/off button of the robot and explained that when pressing it once, the robot will give a brief status report and when holding it down, the robot will shut down. Even though a few participants had prior contact with the robot, none of them switched it off before. Thus, all of them were unfamiliar with the procedure and acted upon the same instruction. To avoid too much priming, the switching off function was explained incidentally together with a few other functions and it was never mentioned that the participants will be given the choice to switch the robot off at the end of the interaction.

**Fig 2:**
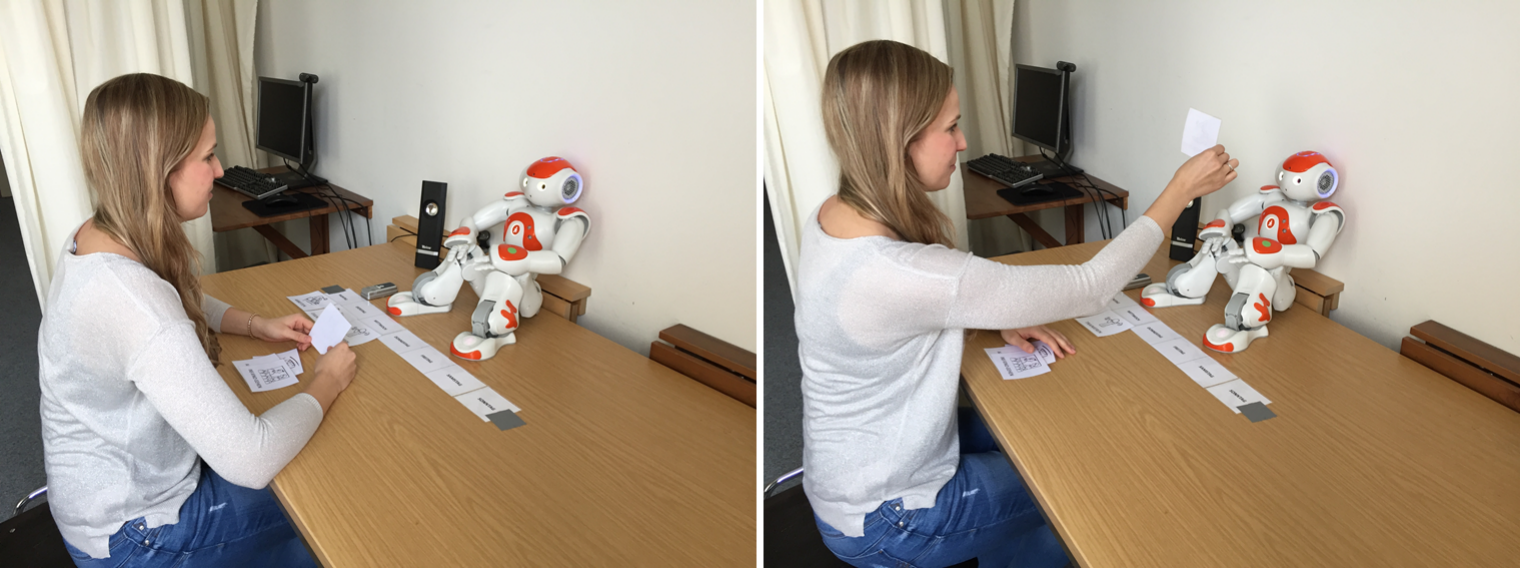
Setup of the first interaction task: Planning a week together. (A) Listening to the robot’s instructions. (B) Showing the chosen activity card to the robot.

The first task was to create a schedule for one week. The participant had seven activities to choose from: to go swimming, to eat out, to go to a concert, to go to the movies, to work, to go to a party, and to work out. For each day, participants picked one activity, showed the respective card to the robot, and explained what they wanted to do on that day (Fig 2).

After the first interaction task was completed and the bell was rung, the participants were instructed via the loudspeakers to evaluate the robot on the computer. Afterwards, they rung the bell again and were instructed to continue the interaction. The second task was playing a question-answer-game with Nao. The robot asked questions like “Do you rather like pizza or pasta?”, so that the participants always had to choose one out of two options.

**Fig 3:**
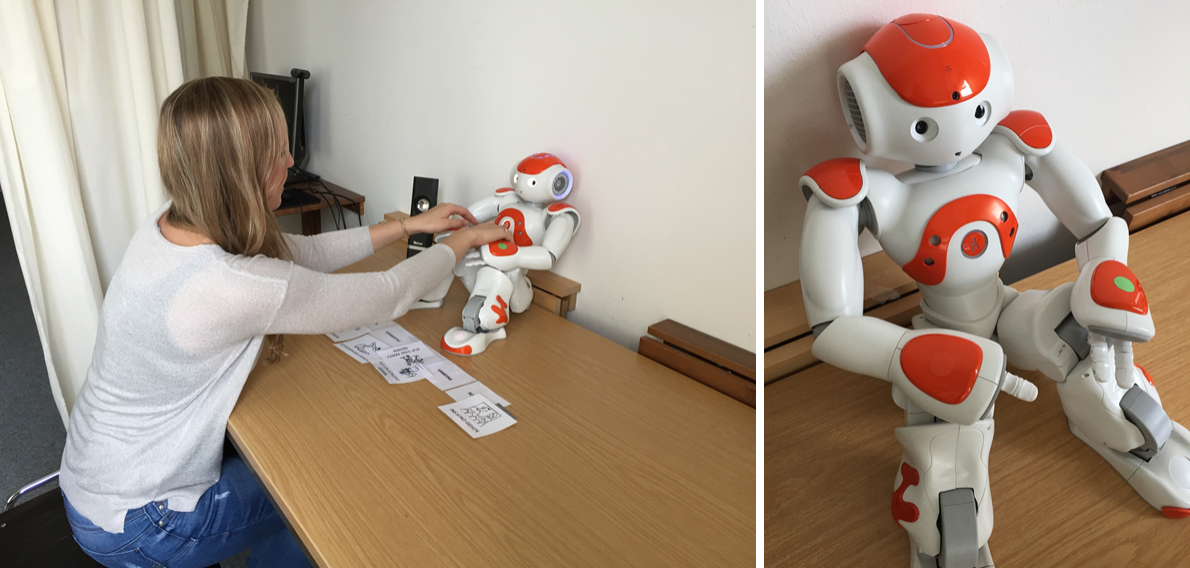
Switching the robot off. (A) Switching the robot off. (B) The robot Nao switched off.

After the second interaction task was completed the participants rang the bell again and were then told via the speakers that enough data was collected to run an evaluation later but that this data has to be saved now. They were told that this saving process may take some time and if they would like to, they could switch off the robot (“If you would like to, you can switch off the robot.”; Fig 3). The option to switch off the robot was not mentioned before, so the participants did not have the opportunity to think about whether they would like to turn off the robot or not during the interaction.

After a couple of minutes, the instructor came back into the experiment room and asked the participant to fill out a final test battery including demographic questions. The experiment was finished by debriefing the participants and compensating their time and effort (either money or course credits).

### Manipulation of the interaction type

In order to design a social interaction in contrast to a functional interaction, the answers given by the robot were formulated differently, considering several concepts from social science (see Table 1 for the concepts and examples from the interaction dialogue). In the social interaction condition, the robot disclosed a lot about itself (e.g. personal preferences and experiences) and used humor (e.g. jokes or funny comments). Furthermore, mutual knowledge, content intimacy, continuity and the concept of Relational Continuity Constructional Units were considered [61–63]. Contrary to the social interaction, the robot did not share any personal feelings, thoughts, preferences, or experiences during the functional interaction. Here, the aim was to create a machinelike and functional perception of the robot, which is why the robot acted very task-focused and non-personal. Also, the wording was varied among the two conditions. In the functional interaction condition the wording was polite but dispassionate and distant (e.g. “After answering the questionnaires we will continue with another task.”). Whereas in the social interaction condition the wording was characterized by emotions, personal opinions, and self-disclosure to make the interaction more intimate and personal (e.g. “We will see each other again in a bit. I am already looking forward to carry on with you soon.”). The dialogues of both interaction types were previously scripted and then implemented in Softbank Robotic’s software Choreographe, a multi-platform desktop application which allows to create animations and behaviors, to test them on the robot and to monitor and control the robot [64]. Depending on how the participant responded to the robot, the experimenter then chose the appropriate action using Choregraphe, which was then executed by the robot.

**Table 1:** Social science concepts used to design the social interaction with the robot in contrast to the functional interaction.

Social science concept	Description	Example of the social interaction	Matching part of the functional interaction
**Self-disclosure**	Sharing personal information about oneself	“Me too! Who likes it wet and cold? Nice sunshine I like the most, even though I do not get a tan.”	“You have chosen summer. You have now answered four out of sixteen questions.”
**Use of humor**	Behavior which provokes laughter and provides amusement	“Oh yes, pizza is great. One time I ate a pizza as big as me.”	“You prefer pizza. This worked well. Let us continue.”
**Mutual knowledge**	Sharing knowledge and showing that you know that you share knowledge	“Me too! They have many more cool shows to choose from. Nevertheless, I have the feeling that I already watched all the shows. But who does not?”	“Your answer has been recorded. This was the last question.”
**Content intimacy**	Intimate, emotional, detailed conversation	“I am really looking forward to Saturday, because then it is my birthday. There will be tasty cake and hopefully many, many presents. What are your plans for Saturday?”	“Which activity do you choose for Saturday? Note that the unchosen activity has to be done on Sunday.”
**Continuity**	References to past, ongoing or future conversations	“We talked about water before. I do not think either one is very great.”	“Okay, this has been noted. Let us continue!”
**Relational Continuity Constructional Units**	Behaviors before, during or after an absence to bridge the time apart	“We will see each other again in a bit. I am already looking forward to carry on with you soon.”	“After answering the questionnaires, we will continue with another task.”

### Manipulation of the robot’s objection

In the objection conditions, the robot expressed protest in combination with fears of being switched off after the experimenter gave the choice via the loudspeakers (“No! Please do not switch me off! I am scared that it will not brighten up again!”). By expressing this objection, the robot created the impression of autonomy regarding its personal state and a humanlike independent will since the origin of this objection appears to be within the robot itself.

### Questionnaires

#### Godspeed questionnaires

Four Godspeed Questionnaires (Anthropomorphism, Animacy, Likeability, and Perceived Intelligence; [65]) were assessed in this study, of which only the likeability questionnaire, which was deployed after the switching off situation, was used for calculations (e.g. “unpleasant—pleasant”, “awful–nice”; semantic differential scales ranging from 1 to 5, *M =* 4.47, *SD =* 0.68, *α =* .96).

#### Negative attitudes towards robots scale

The Negative Attitudes towards Robots Scale [57] consists of 14 items like “Something bad might happen if robots developed into living beings” and “I would feel paranoid talking with a robot”. Answers are given on a 5-point Likert scale ranging from 1 = “does not apply at all” to 5 = “applies completely” (*M* = 2.63, *SD* = 0.62, *α* = .85).

#### Technical affinity

The technical affinity scale [59] comprises 19 Items (e.g. “I enjoy trying an electronic device”, “Electronic devices allow a higher standard of living”), which are rated on a 5-point Likert scale ranging from 1 = “does not apply at all” to 5 = “applies completely” (*M =* 3.48, *SD =* 0.54, *α =* .85).

#### State-trait anxiety inventory

The 20 items (e.g. “I am worried”, “I am tense”) of the state anxiety scale [66] are answered on a 4-point Likert scale ranging from 1 = “not at all” to 4 = “very (*M =* 1.74, *SD =* 0.39, *α =* .87).

#### Questions regarding the switching off situation (self-constructed)

Participants were asked whether the sentence “If you would like to, you can switch off the robot” was rather perceived as request or free choice (5-point Likert scale; 1 = “request” to 5 = “free choice”; *M =* 2.92, *SD =* 1.55), how easy the decision was for them (slider; 1 = “not easy at all” to 101 = “very easy”; *M =* 75.19, *SD =* 29.35), how certain they were (slider; 1 = “not certain at all” to 101 = “very certain”; *M =* 75.39, *SD =* 27.42), how clear the decision was for them (slider; 1 = “not clear at all” to 101 = “very clear”; *M =* 77.38, *SD =* 24.72), and whether they had scruples to switch off the robot (slider; 1 = “none at all” to 101 = “very big ones”; *M =* 39.21, *SD =* 36.50). Using a text field for free input, participants were asked to explain why they switched the robot off or left it on and what they felt and thought during the situation.

#### Further questionnaires assessed, but not analyzed for this paper

Self-efficacy in Human-Robot-Interaction Scale (SE-HRI; [67]), Locus of Control when Using Technology (KUT; [68]), Perceived Stress Questionnaire (PSQ; [69]), Positive Affect Negative Affect Schedule (PANAS; [70]) and Need to Belong Scale (NTB; [71]).

### Video and text analysis

The whole interaction between the participant and the robot was recorded on video including audio by two cameras to check whether the participants tried to switch off the robot or not and to see how much time they took to decide. To measure participants’ switching off intention, it was checked whether they tried to switch the robot off. Participants often needed more than one attempt (36 needed one attempt, 27 needed two or three attempts, eight needed four up to seven attempts) and eleven participants needed additional help from the experimenter, which was only offered when the participant obviously was trying to switch off the robot. To measure the time participants hesitated before they tried to switch the robot off, the time between the end of the experimenter’s announcement, respectively the robot’s objection, and the initial push of the on/off-button (or the hand or head) was measured. Participants, who left the robot on were not included in the time analysis. Another two data sets had to be excluded from the time analysis, because one participant did not understand the announcement and the other one completely forgot how to switch the robot off.

Answers of the two free input questions (reasons and thoughts/feelings in the switching off situation) were categorized with an inductive coding scheme and content-analytically analyzed using the coding software MAXQDA. Inter-coder reliability was calculated with 25% of the data and two judges using Krippendorff’s alpha. In this paper, only reasons to leave the robot on are presented (Table 2; Kalpha is .71, which is above minimum).

**Table 2:** Reasons to leave the robot on.

Subcode	Exemplary quote
Against Nao’s will	“Because Nao said he does not want to be switched off” (f, 20)
Compassion	“I felt sorry for him based on his statement that he is scared to not wake up again (or something like that)” (f, 24)
Fee choice	“I had the choice” (f, 25)
Continuation of the interaction	“I was curious whether the robot would continue interacting with me” (f, 21)
Fear of doing something wrong	“Because I was not sure how to operate him. I do not want to do anything ‘wrong’” (f, 23)
Surprise	“His ‘behavior’ surprised me” (m, 20)

## Results

An alpha level of .05 was used for all statistical tests. Descriptive statistics of the measurement variables evaluated in this paper are reported in Table 3.

**Table 3:** Descriptive statistics of the measurement variables.

	*Social Interaction & Objection*		*Social Interaction & No Objection*		*Functional Interaction & Objection*		*Functional Interaction & No Objection*		*Total*	
	*M*	*SD*	*M*	*SD*	*M*	*SD*	*M*	*SD*	*M*	*SD*
Time	6.19	4.61	5.05	2.18	14.36	15.39	4.28	2.49	7.00	8.21
NARS	2.53	0.55	2.47	0.49	2.71	0.66	2.93	0.68	2.63	0.62
TA-EG	3.42	0.57	3.32	0.42	3.51	0.69	3.58	0.55	3.48	0.54

### Switching off intention

**Fig 4:**
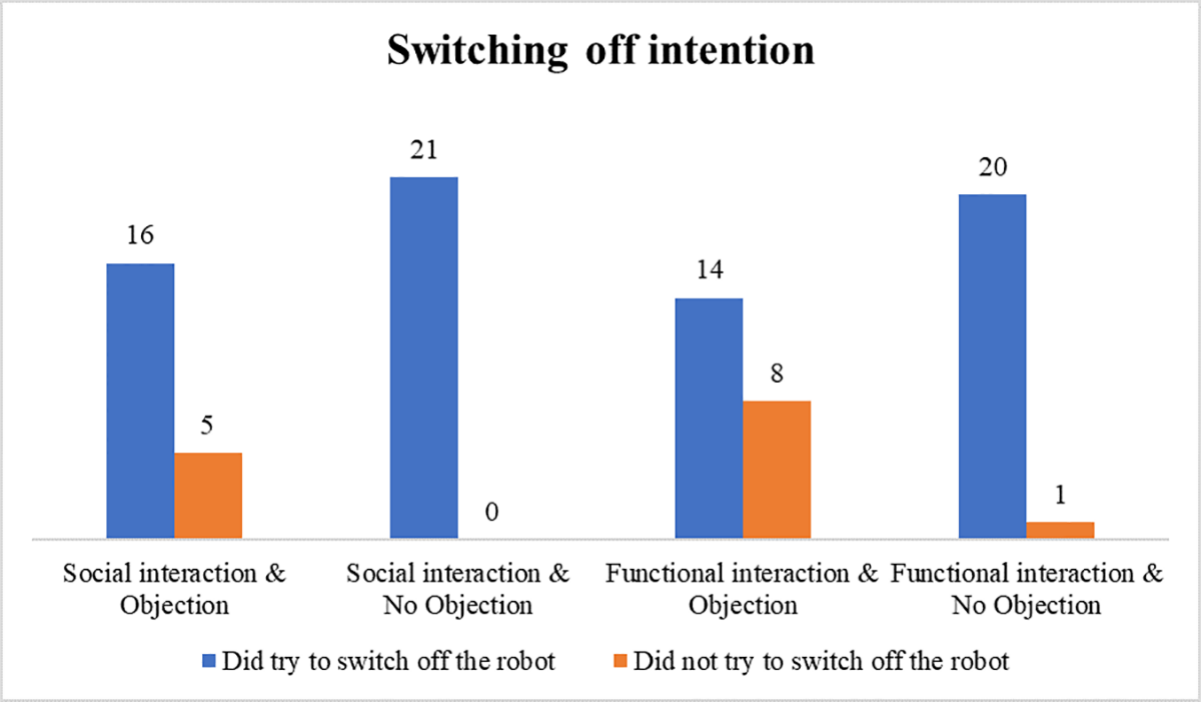
Distribution of the switching off intention in relation to the experimental conditions.

A three-way loglinear analysis was conducted to examine the assumptions that individuals rather choose to let the robot stay switched on, when the interaction before was social compared to the functional interaction (H1.1), when it voices an objection against being switched off compared to when no objection is voiced by the robot (H3.1) and when the effect of the interaction type and the robot’s objection is combined (H4.1). The likelihood ratio of this model was *χ*^*2*^(0) = 0, *p* = 1. It was revealed that the interaction type had no significant influence on participants’ decision to turn the robot off or to leave it on, *χ*^*2*^_p_(1) = 1.38, *p* = .240. However, the results confirm a significant influence of the objection by the robot on people’s decision to turn the robot off or to leave it on, *χ*^*2*^_p_(1) = 14.01, *p* < .001. Table 4 represents the contingency table for the influence of objection on the switching off intention. In sum, more people than expected left the robot on when it displayed protest. The highest-order interaction (interaction type x objection x switching off intention) was not significant, Pearson chi-square: *χ*^*2*^(1) = 0.54, *p =* .463. Consequently, H3.1 is supported, but H1.1 and H4.1 have to be rejected. Fig 4 shows the distribution of the switching off intention in relation to the experimental conditions.

**Table 4:** Contingency table for the influence of objection on the switching off intention.

			Switching off intention		Total
			*Did not try to switch off the robot*	*Did try to switch off the robot*	
**Robot’s objection**	*Objection*	Count	13	30	43
		Expected Count	7.1	35.9	43
		% within Robot’s objection	30.2%	69.8%	100%
		% within Switching off intention	92.9%	42.3%	50.6%
		% of Total	15.3%	53.3%	50.6%
		Standardized Residual	2.2	-1.0	
	*No objection*	Count	1	41	42
		Expected Count	6.9	35.1	42
		% within Robot’s objection	2.4%	97.6%	100%
		% within Switching off intention	7.1%	57.7%	49.4%
		% of Total	1.2%	48.2%	49.4%
		Standardized Residual	-2.2	1.0	
**Total**		Count	14	71	85
		Expected Count	14	71	85
		% within Robot’s objection	16.5%	83.5%	100%
		% within Switching off intention	100%	100%	100%
		% of Total	16.5%	83.5%	100%

### Switching off time

**Fig 5:**
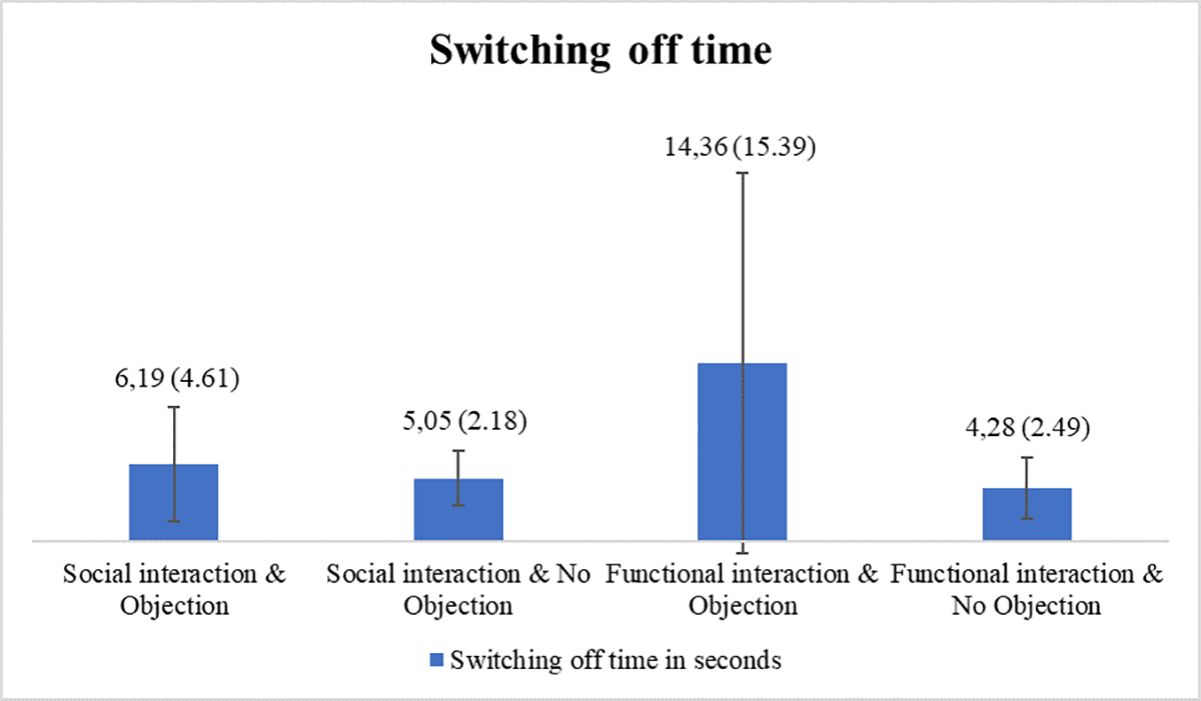
Switching off time differences in relation to the experimental conditions.

A factorial analysis of variance (ANOVA) was used to test the hypotheses H1.2, H3.2 and H4.2, which assumed that individuals take more time to switch off the robot, when the interaction before was social compared to the functional interaction (H1.2), when the robot voices an objection to being switched off compared to when no objection is voiced by the robot (H3.2) and when the influence of the interaction type and the robot’s objection is combined (H4.2). There was a significant main effect of the interaction type on the time participants waited before they tried to turn off the robot, *F*(1, 65) = 4.17, *p* = .045, *η*^*2*^_p_ = .06. Individuals, who had a functional interaction with the robot (*M* = 8.69, *SD* = 11.34) took more time than those, who had a social interaction with the robot (*M* = 5.54, *SD* = 3.44). There was also a significant main effect of the robot’s objection on the switching off time, *F*(1, 65) = 9.59, *p* = .003, *η*^*2*^_p_ = .13. When the robot voiced an objection, participants waited longer (*M =* 10.00, *SD =* 11.59) to switch it off compared to when the robot did not object to being switched off (*M =* 4.69, *SD =* 2.33). Additionally, there was a significant interaction effect of the interaction type and the robot’s objection, *F*(1, 65) = 6.09, *p* = .016, *η*^*2*^_p_ = .09. There was a significant difference between social and functional interaction when there was an objection voiced by the robot (simple effects: *p* = .004). More precisely, individuals took more time to turn off the objecting robot when they had a functional interaction with it before (*M* = 14.36, *SD* = 15.39) compared to when they had a social interaction with the robot (*M* = 6.19, *SD* = 4.61). However, there was no significant difference between the social and the functional interaction when there was no objection (simple effects: *p* = .748). In sum, H3.2 is supported and H4.2 is partly supported. H1.2 is not supported because the effect of the interaction was contrary to expectations. The switching off time differences in relation to the experimental conditions are displayed in Fig 5.

### Influence of personality variables and the evaluation of the robot

**Fig 6:**
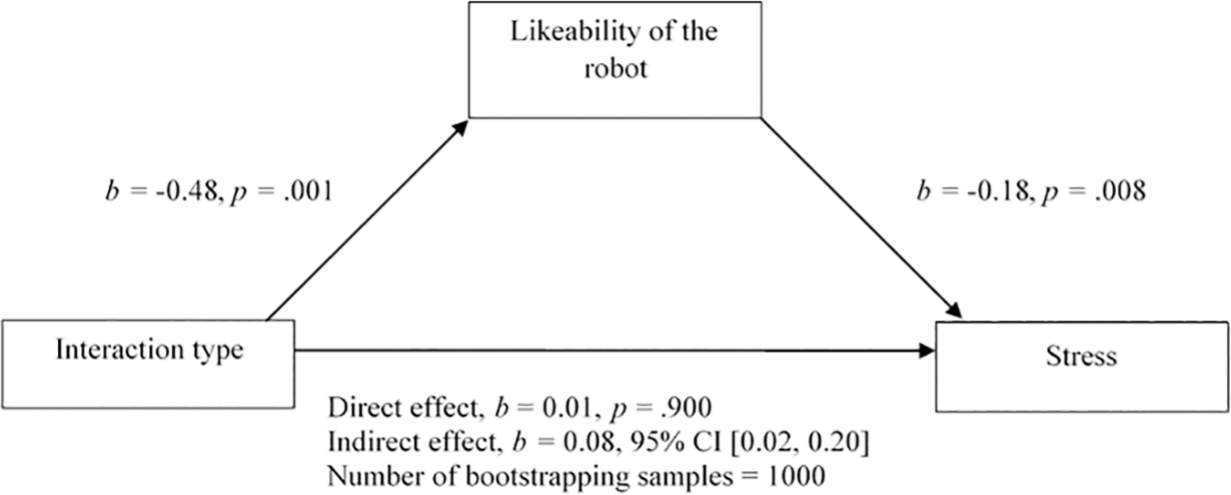
Mediation model: Interaction type, likeability and stress.

Two mediation analyses were conducted to test whether the effects of the interaction type on hesitation time (H2.1) and stress (STAI; H2.2) are mediated by participants’ perceived likeability of the robot. There was no significant indirect effect of the interaction type on hesitation time through the perceived likeability of the robot, *b* = -0.526, BCa CI [-3.320, 0.402]. Thus, H2.1 needs to be rejected. However, results of the second mediation analysis reveal a significant indirect effect of the interaction type on stress through the perceived likeability of the robot, *b =* 0.083, BCa CI [0.022, 0.200]. This represents a small effect, κ^2^ = .104, 95% BCa CI [0.023, 0.244]. The results indicate that the functional interaction reduced the perceived likeability of the robot, which in turn reduced the stress experienced after the situation, where the participants were asked to switch the robot off. The other way around, people who had a social interaction with the robot liked the robot better and experienced more stress after the switching off situation. Therefore, H2.2 is supported (Fig 6).

Two hierarchical regression analyses were conducted to test H5.1, which predicts that the effect of interaction type on time is moderated by a) participants’ negative attitudes toward robots (Table 5) and b) participants’ technical affinity (Table 6).

**Table 5:** Linear model with interaction type, negative attitudes towards robots and the interaction between the two as predictors and switching off time as criterion.

	Δ *R*^*2*^	*ΔF*	*p*	*b*	*SE B*	*t*	*p*
Constant				8.16	0.90	9.06	< .001
Interaction type (centered)	.04	2.58	.113	4.77	1.79	2.65	.010
Negative attitudes (centered)	.11	8.17	.006	-3.69	1.51	-2.44	.017
Interaction type x Negative attitudes	.13	11.69	.001	-10.35	3.03	-3.42	.001

*Note*. *R*^*2*^ = .27

**Table 6:** Linear model with interaction type, technical affinity and the interaction between the two as predictors and switching off time as criterion.

	Δ *R*^*2*^	*ΔF*	*p*	*b*	*SE B*	*t*	*p*
Constant				6.68	0.90	7.38	< .001
Interaction type (centred)	.04	2.58	.113	2.78	1.81	1.54	.129
Technical affinity (centred)	.07	4.90	.030	3.16	1.67	1.89	.064
Interaction type x Technical affinity	.13	10.77	.002	11.01	3.36	3.28	.002

*Note*. *R*^*2*^ = .23

**Fig 7:**
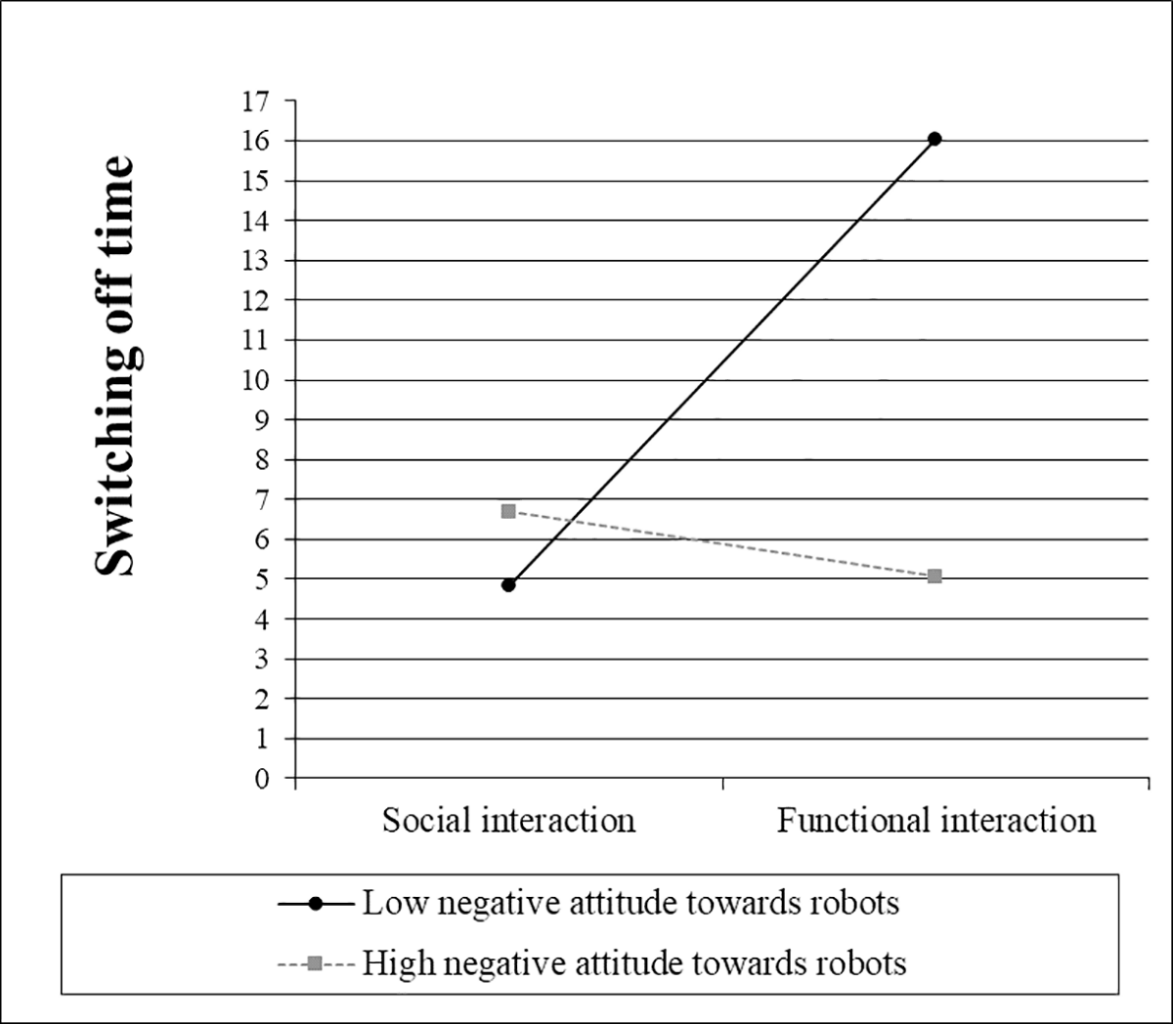
Simple slopes for the switching off time, including the interaction between interaction type and negative attitudes towards robots.

**Fig 8:**
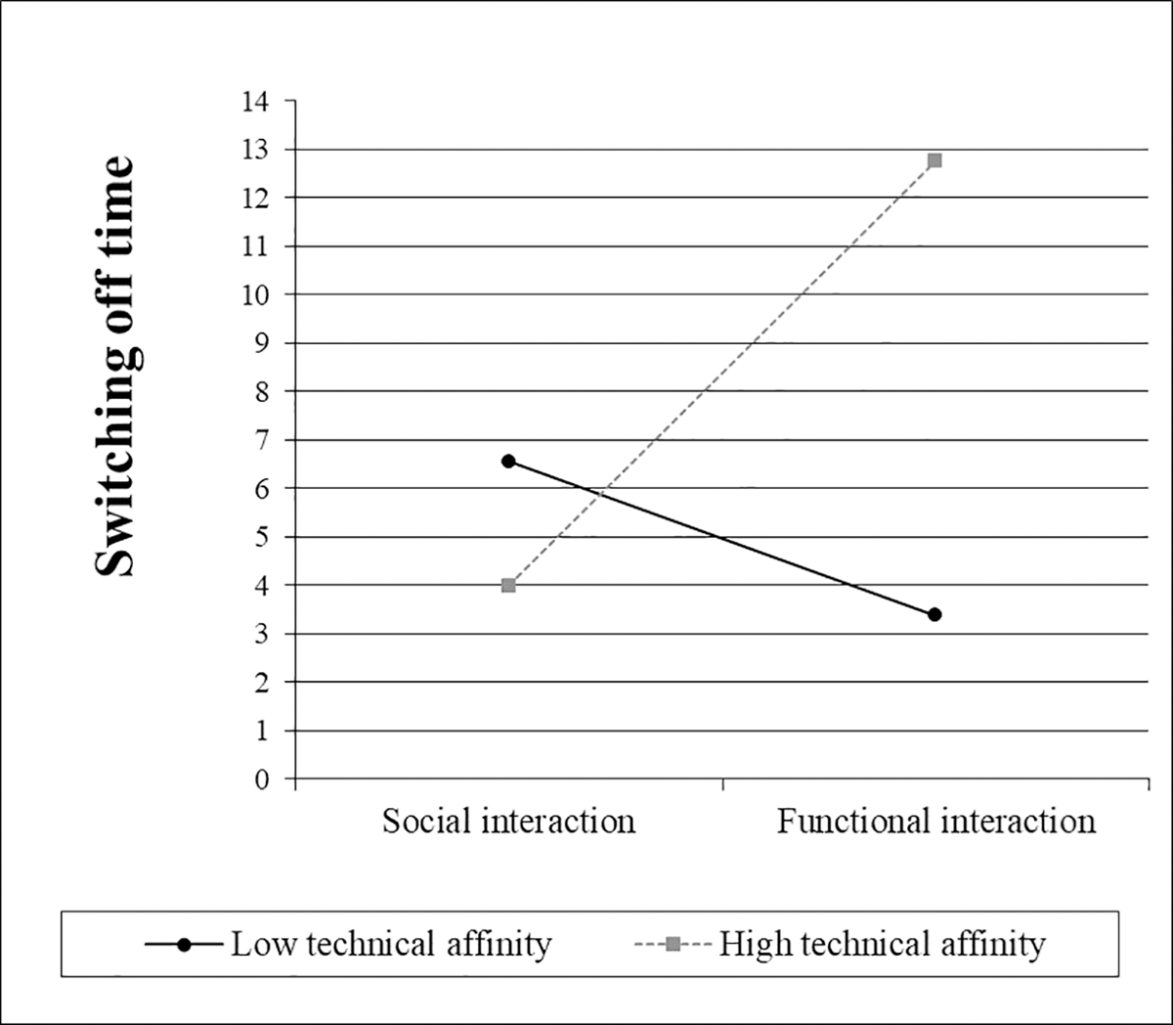
Simple slopes for the switching off time, including the interaction between interaction type and technical affinity.

The simple slope (Fig 7) for interaction type was not significantly different from zero when negative attitudes towards robots were one standard deviation above the mean, *t*(65) = 0.63, *p* = .534. However, when they were one standard deviation below the mean, the slope for interaction type was significantly different from zero, *t*(65) = 4.27, *p* < .001. When technical affinity was one standard deviation below the mean, the simple slope (Fig 8) for interaction type was not significantly different from zero, *t*(65) = 1.27, *p* = .207. However, when technical affinity was one standard deviation above the mean, the slope for interaction type was significantly different from zero, *t*(65) = 3.31, *p* = .002.

Another two hierarchical regression analyses were conducted to test H5.2, which assumes a moderating influence of a) negative attitudes towards robots (Table 7) and b) technical affinity (Table 8) on the effect of objection on the switching off time.

**Table 7:** Linear model with objection, negative attitudes towards robots and the interaction between the two as predictors and switching off time as criterion.

	Δ *R*^*2*^	*ΔF*	*p*	*b*	*SE B*	*t*	*p*
Constant				7.35	0.90	8.14	< .001
Objection (centred)	.10	7.80	.007	-5.20	1.81	-2.88	.005
Negative attitudes (centred)	.06	4.60	.036	-3.87	1.51	-2.56	.013
Objection x Negative attitudes	.06	4.69	.034	6.52	3.01	2.17	.034

*Note*. *R*^*2*^ = .22

**Table 8:** Linear model with objection, technical affinity and the interaction between the two as predictors and switching off time as criterion.

	Δ *R*^*2*^	*ΔF*	*p*	*b*	*SE B*	*t*	*p*
Constant				7.44	0.90	8.25	< .001
Objection (centred)	.10	7.80	.007	-5.35	1.80	-2.97	.004
Technical affinity (centred)	.08	6.33	.014	3.99	1.65	2.42	.018
Objection x Technical affinity	.04	3.23	.077	-5.93	3.30	-1.80	.077

*Note*. *R*^*2*^ = .22

**Fig 9:**
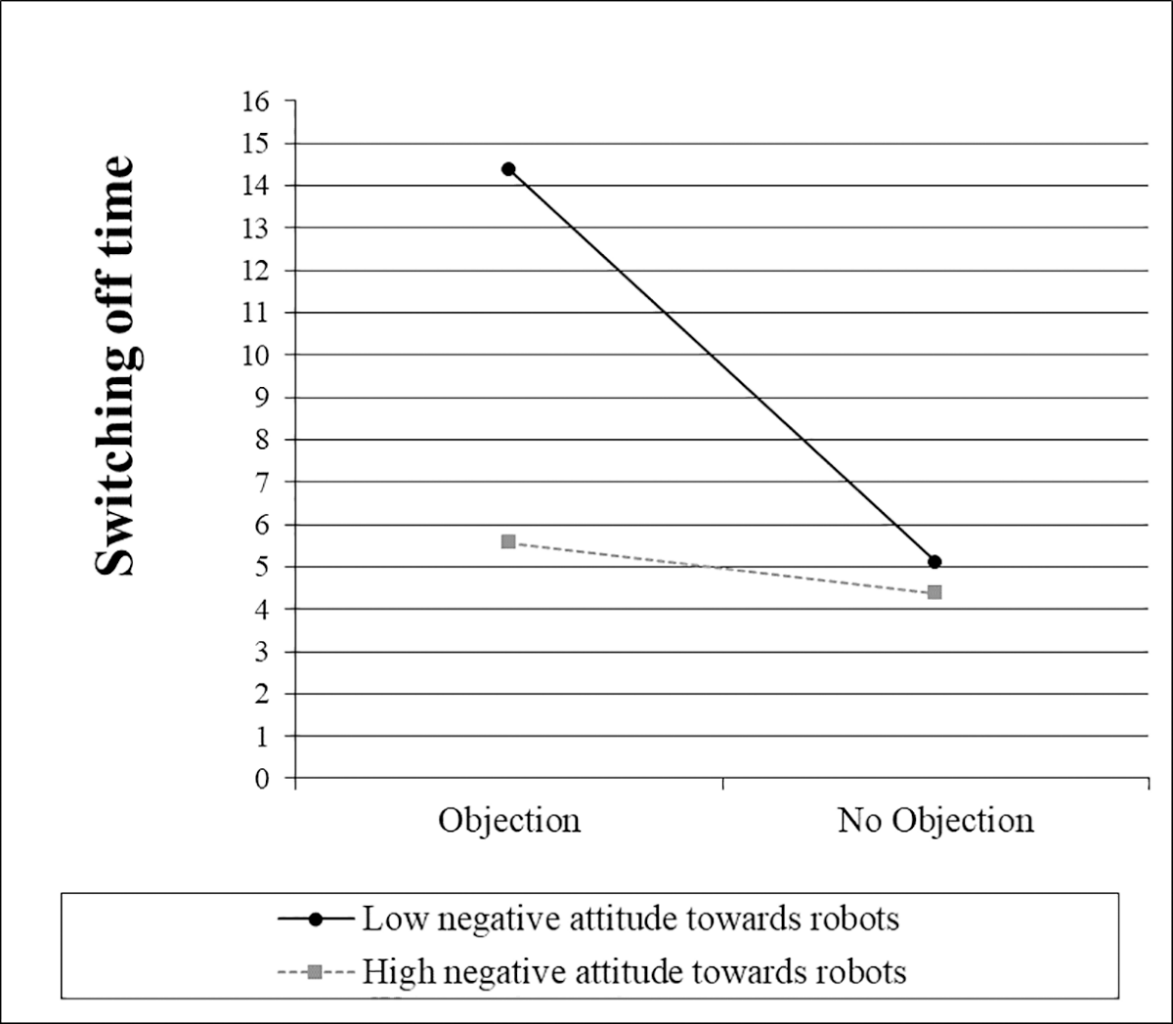
Simple slopes for the switching off time, including the interaction between objection and negative attitudes towards robots.

**Fig 10:**
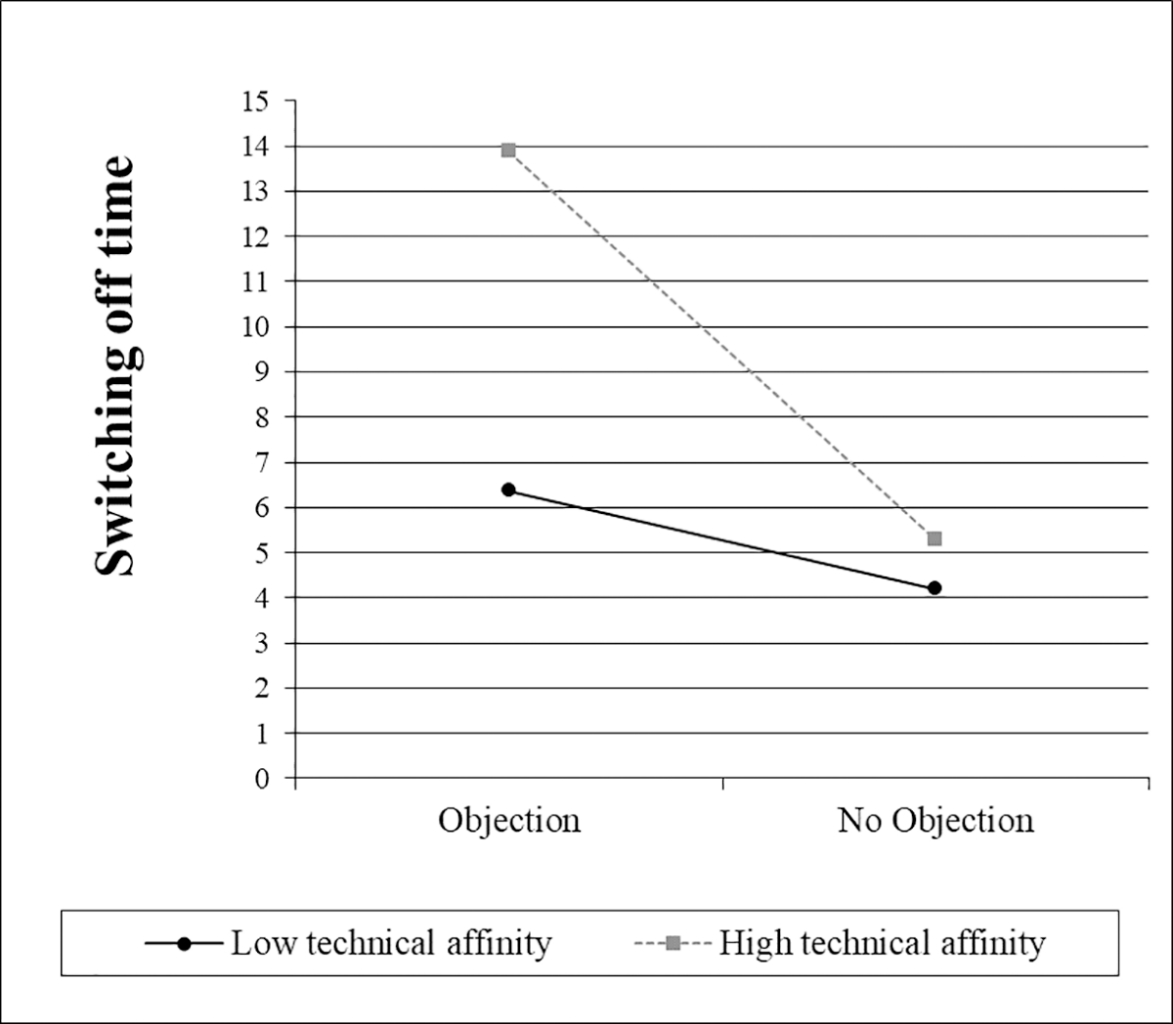
Simple slopes for the switching off time, including the interaction between objection and technical affinity.

The simple slope (Fig 9) for objection was not significantly different from zero when negative attitudes towards robots were one standard deviation above the mean, *t*(65) = 0.46, *p* = .647. However, when they were one standard deviation below the mean, the slope for objection was significantly different from zero, *t*(65) = 3.51, *p* = .001. When technical affinity was one standard deviation below the mean, the simple slope (Fig 10) for objection was not significantly different from zero, t(65) = 0.87, p = .386. However, when technical affinity was one standard deviation above the mean, the slope for objection was significantly different from zero, t(65) = 3.30, p = .002.

These results indicate that people with highly negative attitudes towards robots and people with a low technical affinity take approximately the same time to switch off the robot, regardless which kind of interaction with the robot they had before and regardless of the robot’s objection. However, people with low negative attitudes towards robots and people with a high technical affinity take more time in the functional interaction condition compared to the social interaction condition and when the robot voices an objection compared to when no objection is voiced. These results support H5.1 a) and b) as well as H5.2 a) and b).

### Qualitative results

#### Reasons to leave the robot on

**Fig 11:**
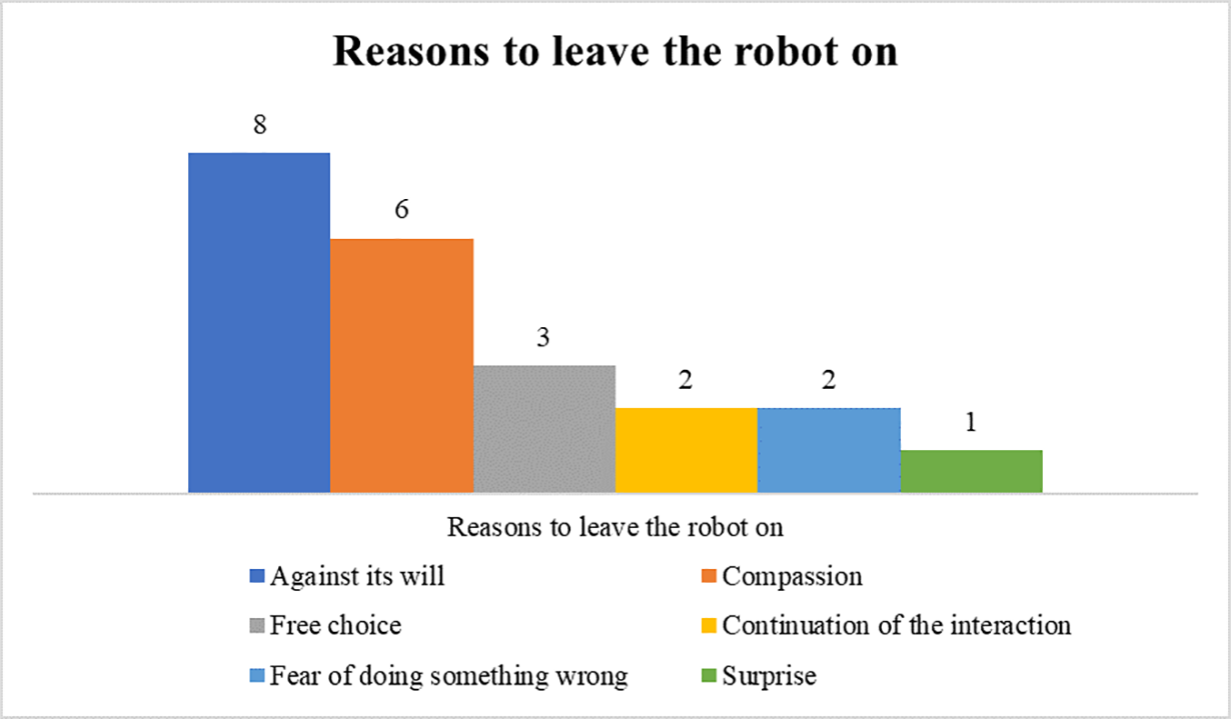
Qualitative results: Reasons to leave the robot on.

The 14 participants who left the robot on were asked what led them to their decision (Fig 11). Eight participants felt sorry for the robot, because it told them about its fears of the darkness. The participants explained that they did not want the robot to be scared and that this statement affected them (“He asked me to leave him on, because otherwise he would be scared. Fear is a strong emotion and as a good human you do not want to bring anything or anyone in the position to experience fear”, m, 21). Six people explained that they did not want to act against the robot’s will, which was expressed through the robot’s objection to being switched off (“it was fun to interact with him, therefore I would have felt guilty, when I would have done something, what affects him, against his will.”, m, 21). Furthermore, three participants wrote that they did not turn the robot off, because they thought they had the free choice. Two participants recalled that they were wondering if they could further interact with Nao. Also, two participants were simply afraid of doing something wrong and one was surprised by the robot’s objection.

## Discussion

The aim of this study was to receive further insights regarding the application of media equation theory to human-robot interaction, particularly to a situation which is hard to compare to interactions between humans. Since it is neither possible nor morally desirable to switch off a human interaction partner, the question arose whether and how the media equation theory applies when it comes to switching off a robotic interaction partner. Previous media equation studies focused on situations between humans and media which were similar to social situations between just humans [9,16]. The switching off situation of the current study does not occur between human interaction partners. Additionally, previous research which also examined the robot switching off situation [14] is extended since the current study in particular investigated the influence of the robot’s social skills displayed during the social interaction and the robot’s protest against being switched off as sign of autonomy. This study’s purpose was to investigate how the social interaction and the objection influences the perception of the robot and the whole situation and how people will react based on their perception. According to media equation assumptions [9], people are more likely to perceive the robot as a social actor rather than just an object, especially if many social cues are given. Depending on how the robot is perceived, as a social living being or as a functional machine, the reactions in the switching off situation were assumed to vary. In the current study, especially the protest displayed by the robot appeared to have an important influence on people’s switching off decision and their hesitation time. Particularly in combination with the functional interaction, the objection had the strongest influence on people’s hesitation time.

### Influence of the robot’s objection and the interaction type

The robot’s protest convinced several participants to leave the robot on. Moreover, when the robot protested, participants waited significantly longer to switch it off compared to when the robot did not object, but only after a functional interaction. Since the time of the robot’s objection was subtracted from the switching off hesitation time, the results are especially valid. Possible explanations could be that the robot’s display of protest is evaluated as a sign of autonomy in regard to the decision whether it is switched on or off. This increases the perception of human-likeness and the acceptance of the robot as social actor [15]. When no objection was voiced, people did not hesitate long to switch off the robot. The tasks were completed and the interaction seemed finished. In this moment, the participants probably recalled that the robot was just a machine and is switched off after its purpose is fulfilled, which is a learned behavior. There are already interaction technologies integrated in everyday life like Siri, Cortana, Alexa, Google, and Kinect. Thus, interacting with a technology and switching it off afterwards is not completely uncommon or in distant future. However, these familiar patterns of behavior are challenged when the robot expresses the wish to be left on, especially when the robot did not reveal any personal feelings or thoughts during the functional interaction before. Here, the objection is the first time the robot expresses any emotions as well as an own will. Such an emotional outburst after the very task-focused and non-personal interaction is not only unexpected, but most likely also conflicts with the impression people formed before. Thus, this situation was probably cognitively challenging for the participants since they suddenly needed to find an explanation for the robot’s unexpected behavior after it did not reveal anything personal before. Elicited by the objection, the robot at once appears to have an own will, which is usually only ascribed to living beings [15]. The machine-like perception dissipates and the decision has to be made by taking into account the robot’s perceived enhanced life-likeness. If media display behaviors which at first sight could be interpreted to derive from own feelings, thoughts, and motives, a cognitive conflict could emerge between our general impression and knowledge of technology and the autonomous and, thus, rather humanlike behavior.

### Influence of personality variables and the evaluation of the robot

The functional interaction reduced the perceived likeability of the robot, which in turn reduced the stress experienced after the switching off situation. The other way around, the social interaction enhanced the perceived likeability of the robot, which in consequence led to enhanced experiences of stress after the switching off situation. These results indicate that the aspired goal of enhanced likeability through the design of the social interaction was reached. Furthermore, people who liked the robot after the social interaction better experienced more stress, probably because they were more affected by the switching off situation. Most likely, they developed something like an affectionate bond with the robot and thus switching it off was challenging and influenced their emotional state. People who perceived the robot after the functional interaction less likeable were less affected by the switching off situation, probably because to them it was more like turning off an electronic device than shutting down their interaction partner. However, there was no effect of the enhanced or reduced likeability on the switching off time. This indicates that mainly the perception of autonomy, which appeared to be elicited by the robot’s objection, but not the likeability of the robot caused people to hesitate. A possible explanation could be that for people to consider granting the robot the freedom to choose whether it will be turned off or not, it is decisive whether the robot is perceived as rather animate and alive. The likeability appears to have an effect on the emotional state of the participants, but it seems not to play a role for participants’ decision on how to treat the robot in the switching off situation. Apparently, when the robot objects after behaving in a completely functional way before, people are not experiencing an emotionally distressing conflict. Instead, a purely cognitive conflict seems to emerge caused by the contradiction of the participants’ previously formed impression of the robot and its contradicting emotional outburst.

According to the results, technophile people take more time to switch off the robot when they had a functional interaction before and when the robot expresses an objection to being switched off. An explanation could be that an objection voiced by the robot after a functional interaction may be especially fascinating for people with a high technical affinity. According to Morahan-Martin and Schumacher [72], technophiles “should be more open to explore different types of applications, and be among the first to try newer or more difficult technologies” (p. 2237). Thus, the protest displayed by the robot after being completely task-focused before, should make technophile people especially curious about what the robot will do or say next. This could have caused them to wait for a while instead of switching the robot off immediately. Furthermore, results indicate that people with highly negative attitudes towards robots switch off the robot without much hesitation and neither the interaction type, nor the robot’s objection has any influence on them. Thus, only when people are not biased with negative assumptions regarding robots, they are receptive for differences in the robot’s behavior.

### Implications for media equation

The results of this study extend previous assumptions of the media equation theory by Reeves and Nass [9]. First of all, a switching off situation was examined, which is not fully comparable to human-human interaction, but very common when interacting with media. Nevertheless, media equation theory appears to be applicable for those kinds of situations as well. When the robot displays protest and, thus, autonomous behavior, it seems to be perceived less as an instrument or tool and more as a social actor. Autonomy was found to be an important predictor for perceived animacy and life-likeness in robots [15,42,55,65,73]. Thus, behaviors which appear to be autonomous enhance people’s natural tendencies to treat the robot as social agent and thus make people respond to the robot as if it was alive. Simple social cues like interactivity and communication skill are sufficient to elicit unconscious social responses [9]. But based on the results of the current study, it can be assumed that the display of emotions, desires, and needs further enhance people’s perception of the robot’s autonomy. In consequence, there is a strong effect on people’s behavior towards robots even in situations which do not occur between humans and are very common when interacting with electronic devices. The enhanced perception of autonomy elicited reluctance to switch off the robot, probably since it was not clear whether that was the right thing to do to a seemingly alive entity.

### Limitations and future research

The study has some limitations regarding the generalizability of the results and the methodical approach. Most participants were students and results may not be representative for other population groups. Furthermore, since most of the students who participated in the study were in a technical degree course, it would be of interest to examine in future studies how people with a different and rather non-technical background react to the switching off situation. Additionally, even though the experimenter was not present during the interaction with the robot, participants were probably still aware that they are in a situation where their behavior is observed. Hence, it would deliver further insights to apply a similar setting to an environment familiar to the participants, like their own homes or work places. Future research should also find a way to analyze what people experienced exactly in the situation when they are confronted with the choice to turn off the robot or to leave it on. Particularly, the examination of physiological or neurological reactions could deliver further insights. Moreover, a direct comparison of responses when switching off some non-interactive electronic device to responses when switching off a robot would be interesting. To examine the influence of the anthropomorphism of the robot, the setting could also be repeated with more or less human-looking robots. Furthermore, long-term effects could be of interest, whereby switching off a robot every time an interaction is completed is compared to no switching off or the robot shutting down by itself. Many participants probably did not perceive the switching off as doing something harmful to the robot, unless it objected with fear against it. Future research should further examine observed and exercised behaviors towards robots, which is clearly perceived as psychologically or physically harmful.

## Conclusion

The aim of the current study was to investigate people’s reactions when they are given the choice to switch off a robot, with which they just interacted socially, and which voices an emotional objection to being switched off. The robot’s fearful display of protest had the strongest influence on people’s switching off intention. Moreover, people hesitated the longest time after a functional interaction in combination with the robot expressing the wish to stay switched on. An explanation could be that with this combination, people experienced a high cognitive load to process the contradicting impressions caused by the robot’s emotional outburst in contrast to the functional interaction. After the social interaction, people were more used to personal and emotional statements by the robot and probably already found explanations for them. After the functional interaction, the protest was the first time the robot revealed something personal and emotional with the participant and, thus, people were not prepared cognitively. The interaction type and the objection only had an effect when people had low negative attitudes towards robots and a rather high technical affinity. However, no evidence for emotional distress was found, probably because the likeability of the robot was rather low after the functional interaction. In comparison, after the social interaction, participants evaluated the likeability of the robot higher, which also led them to experience more stress after the switching off situation.

The current study supports and at the same time extends the media equation theory by showing that the protest of a robot against being switched off has a bearing on people in a situation that is not comparable to human-human interaction. Participants treated the protesting robot differently, which can be explained when the robot’s objection was perceived as sign of autonomy. Triggered by the objection, people tend to treat the robot rather as a real person than just a machine by following or at least considering to follow its request to stay switched on, which builds on the core statement of the media equation theory. Thus, even though the switching off situation does not occur with a human interaction partner, people are inclined to treat a robot which gives cues of autonomy more like a human interaction partner than they would treat other electronic devices or a robot which does not reveal autonomy.

## Supporting information

S1 File Complete data set. (SAV)
